# Association between nitric oxide synthase T-786C genetic polymorphism and chronic kidney disease: Meta-analysis incorporating trial sequential analysis

**DOI:** 10.1371/journal.pone.0258789

**Published:** 2021-10-18

**Authors:** Po-Jen Hsiao, Chih-Chien Chiu, Dung-Jang Tsai, Pi-Shao Ko, Ying-Kai Chen, Hao Cheng, Wen Su, Kuo-Cheng Lu, Sui-Lung Su

**Affiliations:** 1 Division of Nephrology, Department of Internal Medicine, Taoyuan Armed Forces General Hospital, Taoyuan, Taiwan, R.O.C.; 2 Division of Nephrology, Department of Internal Medicine, Tri-Service General Hospital, National Defense Medical Center, Taipei, Taiwan, R.O.C.; 3 Department of Life Sciences, National Central University, Taoyuan, Taiwan, R.O.C.; 4 Big Data Research Center, Fu-Jen Catholic University, New Taipei City, Taiwan, R.O.C.; 5 Division of Infectious Diseases, Department of Internal Medicine, Taoyuan Armed Forces General Hospital, National Defense Medical Center, Taoyuan, Taiwan, R.O.C.; 6 School of Public Health, National Defense Medical Center, Taipei, Taiwan, R.O.C.; 7 Graduate Institute of Life Sciences, National Defense Medical Center, Taipei, Taiwan, R.O.C.; 8 Division of Nephrology, Department of Medicine, Zuoying Branch of Kaohsiung Armed Forces General Hospital, Kaohsiung, Taiwan, R.O.C.; 9 Graduate Institute of Aerospace and Undersea Medicine, National Defense Medical Center, Taipei, Taiwan, R.O.C.; 10 Division of Nephrology, Department of Medicine, Fu-Jen Catholic University Hospital, School of Medicine, Fu-Jen Catholic University, New Taipei City, Taiwan, R.O.C.; Shanghai Jiao Tong University, CHINA

## Abstract

**Background:**

Several meta-analyses of the relationship between endothelial nitric oxide synthase (eNOS) T-786C gene polymorphism and chronic kidney disease (CKD) have been published. However, the results of these studies were inconsistent, and it is undetermined whether sample sizes are sufficient to reach a definite conclusion.

**Objective:**

To elucidate the relationship between T-786C and CKD by combining previous studies with our case-control sample and incorporate trial sequential analysis (TSA) to verify whether the sample size is adequate to draw a definite conclusion.

**Methods:**

PubMed and Embase databases were searched for relevant articles on eNOS T-786C and CKD before February 28, 2021. TSA was also incorporated to ascertain a conclusion. A total of 558 hemodialysis cases in the case-control study was recruited from nine dialysis centers in the northern area of Taiwan in 2020. Additionally, 640 healthy subjects of the control group, with estimated glomerular filtration rate (eGFR) ≥ 60 mL/min/1.73 m^2^, were selected from participants of the annual elderly health examination program at the Tri-Service General Hospital. The functional analysis was based on eQTL data from GTExPortal.

**Results:**

After screening with eligibility criteria, 15 papers were included and eventually combined in a meta-analysis. The result of the TSA showed that the sample size for Caucasians was adequate to ascertain the correlation between eNOS T-786C and CKD but was insufficient for Asians. Therefore, we added our case-control samples (n = 1198), though not associated with CKD (odds ratio [OR] = 1.01, 95% confidence interval [CI] = 0.69–1.46), into a meta-analysis, which supported that eNOS T-786C was significantly associated with CKD in Asians (OR = 1.39, 95% CI = 1.04–1.85) by using an adequate cumulative sample size (n = 4572) analyzed by TSA. Data of eQTL from GTEx showed that T-786C with the C minor allele exhibited relatively lower eNOS mRNA expression in whole blood, indicating the hazardous role of eNOS T-786C in CKD.

**Conclusions:**

eNOS T-786C genetic polymorphism was of conclusive significance in the association with CKD among Asians in our meta-analysis. Our case-control samples play a decisive role in changing conclusions from indefinite to definite.

## 1. Introduction

The global prevalence of chronic kidney disease (CKD) is 13.4% [1]. As renal function decreases, CKD will gradually develop into end-stage renal disease (ESRD). Patients with ESRD must receive renal replacement therapy, causing them to shoulder high medical costs and to have decreased quality of life [2]. Therefore, examining the risk factors for kidney disease is important. Currently, known CKD risk factors include genetic factors, diabetes, hypertension, and family history [3]. Studies have also revealed that many gene polymorphisms will affect the risk of developing CKD [4,5].

The etiology of diabetes, hypertension, and other diseases related to vascular endothelial dysfunction and nitric oxide (NO) concentrations will affect vascular endothelial function [6]. NO is a gaseous, lipophilic molecule whose main function is to dilate the afferent and efferent arterioles of the kidneys, increase glomerular filtration rate, and promote sodium excretion by the kidneys [7]. Changes in vascular NO concentrations will also affect the risk of developing CKD [8]. NO is synthesized by endothelial NO synthase (eNOS) expressed in the vascular endothelium, which inhibits vascular inflammation, controls vascular smooth muscle proliferation, and stimulates angiogenesis [9].

Several papers have examined the relationship between eNOS gene polymorphisms and CKD, of which commonly examined loci include 4b/a in intron four, G894T in exon seven, and T-786C in the promoter region [10]. Previously, many studies have studied the relationship between intron 4b/a and G894T with CKD, and definite conclusions were obtained [11–15]. A study showed that the polymorphism in T-786C would reduce gene promoter activity and decrease eNOS translation. This will cause impairment of vascular NO synthesis and increase the risk of developing kidney disease, arteriosclerosis, and other diseases [11].

Several meta-analyses have examined the relationship between eNOS T-786C and CKD [16–18] but generated inconsistent results. For example, some studies have found that the presence of the C allele at eNOS T-786C correlates with diabetic nephropathy [18]. By contrast, other studies have found that this gene polymorphism is not related to ESRD risk [16]. The latest meta-analysis article was published in 2015 and included 4203 subjects. However, many new case-control studies are still excluded in that meta-analysis [19–21]. Additionally, previous meta-analyses were unable to prove if a definite conclusion can be ascertained from the correlation between eNOS T-786C and CKD [22].

This study employed a meta-analysis to elucidate the relationship between eNOS T-786C gene polymorphisms and CKD and incorporated trial sequential analysis (TSA) to verify whether a definite association can be confirmed by adding our case-control samples to previous literature.

## 2. Materials and methods

### 2.1 Meta-analysis

#### 2.1.1 Search methods and criteria for study consideration

The PRISMA checklist and meta-analysis on genetic association studies checklist are described in S1 Table [23]. The study subjects were sampled from the general population. Synonymous words of “eNOS T-786C” and “chronic kidney disease” were used to search the PubMed and Embase databases for articles up to February 28, 2021 (S2 Table), and the language was limited to English. Additionally, we manually scanned the reference list of individual papers included in previous meta-analyses to avoid the omission of essential articles. The inclusion and exclusion criteria were as follows: (1) Case-control studies or cross-sectional studies were included. (2) There must be a clear diagnosis of case groups, such as proteinuria, low glomerular filtration rate, and other abnormalities in renal function biomarkers, or parenchymal damage in the kidney as diagnosed through biopsy, computed tomography, ultrasonography, and other examinations. Patients with lupus nephritis, polycystic kidney disease, endemic nephropathy, and reflux nephropathy were excluded. (3) Subjects in the control group must have normal renal function. (4) The article should contain the genetic distribution of target loci. (5) Adults aged > 18 years. (6) Papers that do not have complete genetic information were removed.

#### 2.1.2 Data extraction

In this study, two reviewers (Po-Jen Hsiao and Cheng Hao) were responsible for the independent extraction of literature data. The data extracted include the last name of the first author, year of publication, country, ethnicity of the study population, and detailed genetic distribution in the case and control groups.

#### 2.1.3 Statistical analysis

All included papers were described using appropriate proportions or mean values. Our meta-analysis uses odds ratio [ORs] with 95% confidence interval [CI] to examine the relationship between eNOS T-786C and CKD. The I^2^ test was used to assess heterogeneity. I^2^ > 50% indicates the presence of a moderate to high heterogeneity. Different genetic models were used to calculate the risk of CKD for eNOS T-786C under random-effects model assumptions. Egger’s test and funnel plot were used to detect publication bias. The significance level was set as 0.05. Statistical analysis and data visualization were conducted using R software v.3.3.1 and “metafor” [24] and “meta” [25] packages.

#### 2.1.4 TSA

This study used TSA to validate whether the results of the meta-analysis could reach definite conclusions [26]. The settings of parameters were listed as follows. The number of patients with the C allele was inputted into the intervention group, the number of patients with the Group T allele was inputted into the control group, the number of CKD patients was inputted into events, and the sum of CKD and healthy subjects was inputted into the total number. With regard to the boundary, the type 1 error was set as 0.05. Regarding sample size estimation, a model variance-based preset value was selected for heterogeneity, and power was set to 0.80. According Sholom *et al*. [27], an OR of 1.5 is a reasonable value for the relationship between genes and diseases. Since the C allele may be a possible risk factor, the OR was set as 1.5. For gene frequency (minor allele frequency), the definitions by the 1000 Genomes for Taiwan as used in Asians and Caucasians were set to 0.10 and 0.23, respectively.

### 2.2 Case-control study

#### 2.2.1 Ethical issues

This study was approved by the Institutional Review Board of the Tri-Service General Hospital (TSGH), a medical teaching hospital of the National Defense Medical Centre in Taipei, Taiwan (TSGH-1-104-05-006). Volunteers signed the informed consent form after the investigators have explained the study.

#### 2.2.2 Subjects

Subjects in the case group were selected from nine dialysis clinics in the northern area of Taiwan in 2020. All hemodialysis patients were diagnosed by professional nephrologists since 2000. This study included patients with the following criteria: (1) dialysis period of less than 3 months, (2) cancers, (3) and insufficient blood samples. Finally, 558 cases were included in the study analysis.

Subjects in the control group were selected from volunteers who participated in the annual elderly health examination program at the Health Management Centre of TSGH. Serum creatinine concentration was acquired through a biochemistry blood test and transformed into eGFR using the MDRD formula [28]. Therefore, subjects with the following criteria were excluded: (1) eGFR < 60 ml/min/1.73 m^2^, (2) kidney diseases (such as positive for proteinuria), (3) cancers, and (4) insufficient volume of blood samples. Finally, 640 subjects were included in the control group for analysis.

Demographic data included age, gender, history of diabetes, hypertension, body mass index (BM in kg/m^2^), and the results of the blood biochemistry examination (i.e., blood urea nitrogen, creatinine, triglycerides, cholesterol, and eGFR) were collected from the questionnaire and medical records.

#### 2.2.3 Genomic DNA extraction and genotyping

Medical technologists or nurses collected 5 ml of intravenous blood samples from volunteers. Genomic DNA was extracted using standard procedures for proteinase K (Invitrogen, Carlsbad, CA, USA) digestion. Additionally, inter-replication validation was used to assess genotyping quality. Inter-replication validation was repeated for 78 samples (approximately 10%), and the concordance rate was 100%.

#### 2.2.4 Statistical analysis

Continuous variables in the general demographic data were expressed as mean ± SD in Student’s t-test. The control group was tested for representativeness using the Hardy–Weinberg equilibrium test. The differences in genotypes and allelic frequencies between hemodialysis patients and healthy controls were tested using χ^2^ test or Fisher’s exact test. The ORs and 95% CIs for the risk of ESRD were calculated using logistic regression. Calculation of genetic polymorphism and the risk of ESRD was expressed using the allele, co-dominant, and dominant/recessive models. *P* < 0.05 was regarded as significant, and the Bonferroni correction was used for multiple corrections. R v.3.3.1 (R Project for Statistical Computing, Vienna, Austria) was used as statistics software.

## 3. Results

### 3.1 Meta-analysis

**Fig 1:**
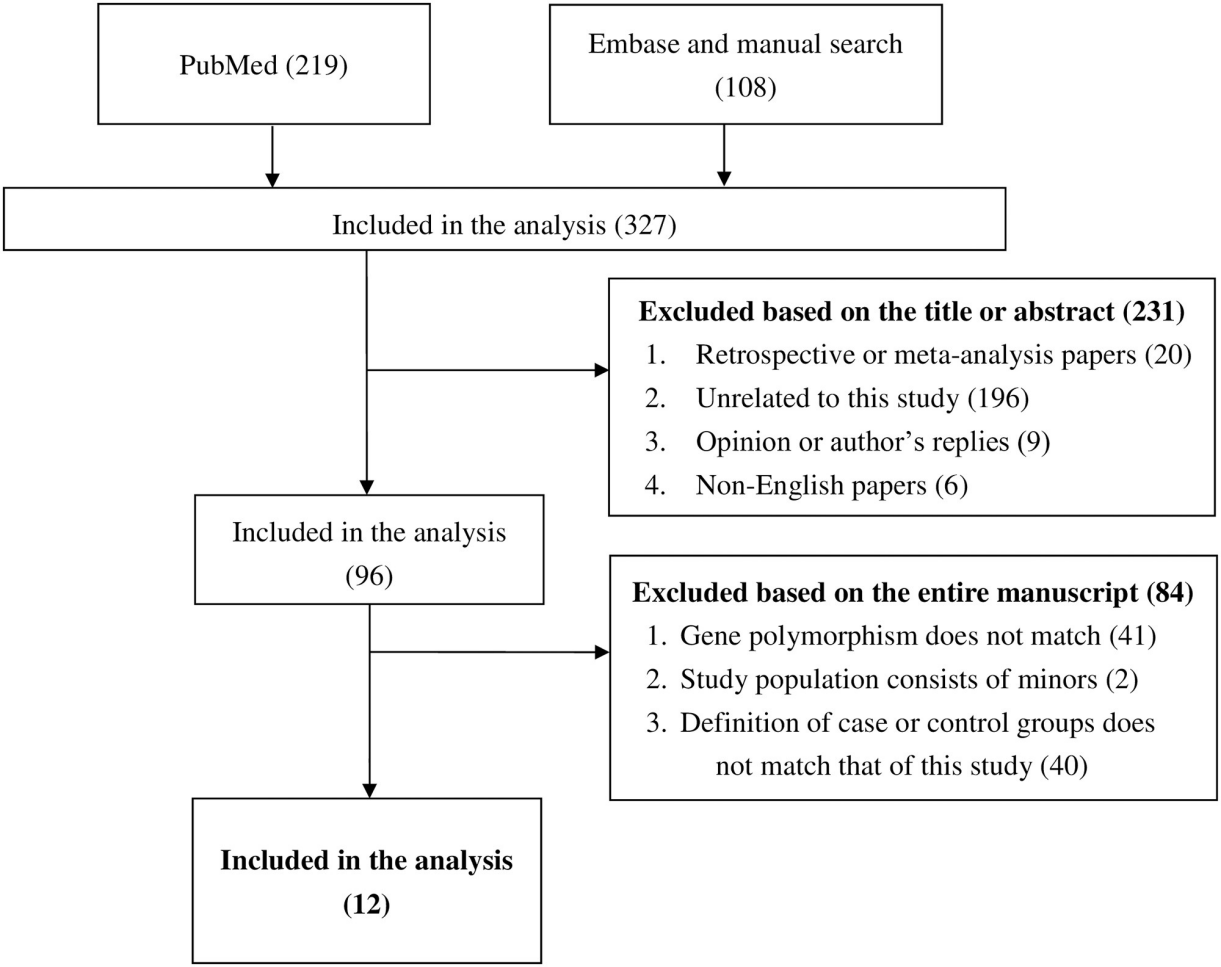
Flow diagram of the identification process for eligible studies.

Fig 1 shows the literature searching flowchart of this study. We first searched 240 articles from PubMed, obtained another 134 reports from Embase, and manually searched 374 articles from the reference list of other meta-analyses. After screening titles and abstracts, 30 retrospective or meta-analysis articles, 230 articles that were unrelated, nine commentaries, and seven non-English articles were excluded. Moreover, 41 articles that were not discussing eNOS T-786C, 40 articles with definitions of case or control groups do not match our inclusion criteria, and two articles with study samples were also excluded. Finally, 15 papers were included in the quantitative synthesis. The Supplementary Table demonstrates the general characteristics of the articles included in the meta-analysis.

**Fig 2:**
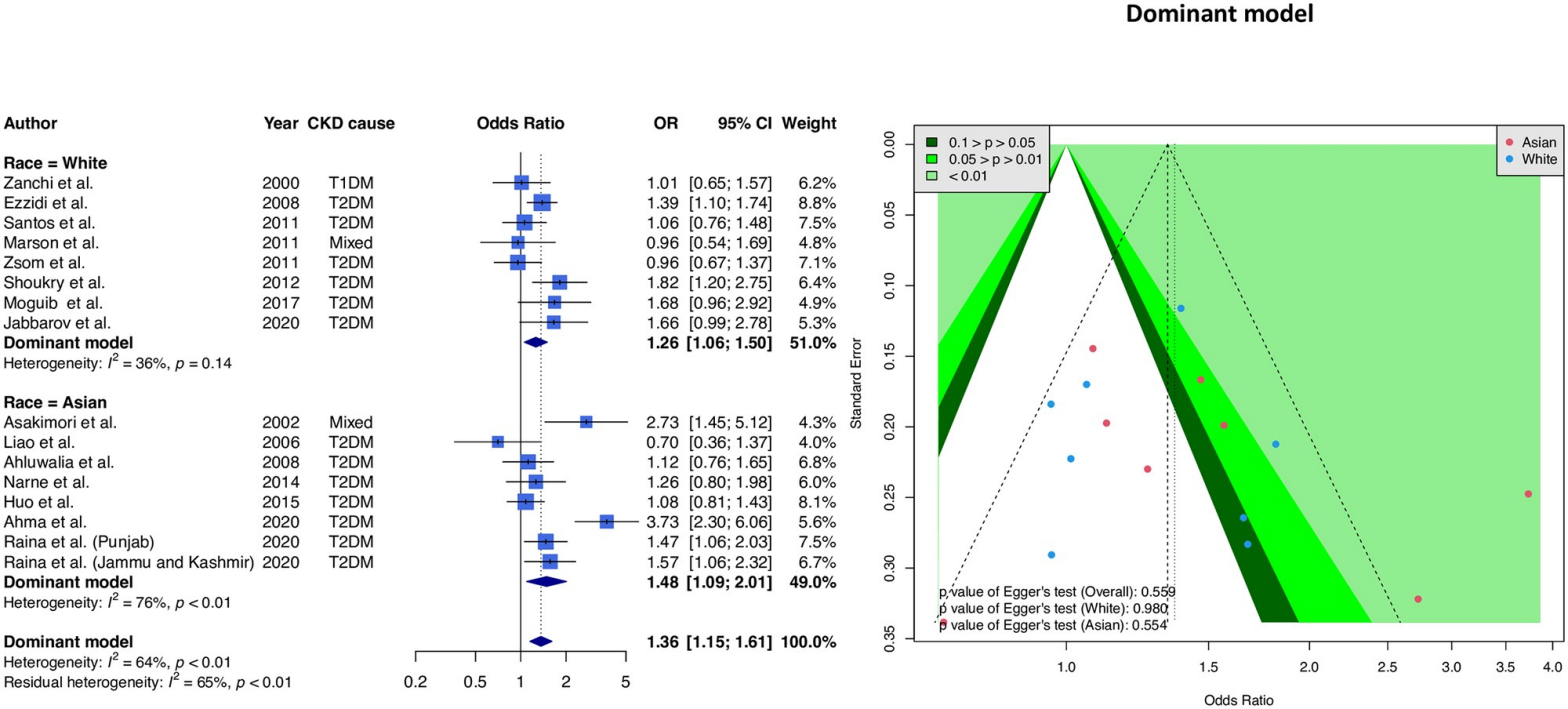
Forest and funnel plots of the relationship between eNOS T-786C alleles and CKD (dominant model). The forest plot is based on the dominant model assumption (reference: TT genotype), and the funnel plot is based on the dominant model assumption; we found a significant association among the Asian and White subgroups. However, the funnel plot indicated balanced symmetry in this meta-analysis.

This study used the dominant model (C/C or C/T genotypes *vs*. T/T genotype) to combine 16 samples. Fig 2 shows the forest and funnel plots. The combined result of all articles showed a significantly higher risk of CC and CT genotypes (OR = 1.36, 95% CI = 1.15–1.61). Ethnicity-stratified results showed a significant association for Asians (OR = 1.48, 95% CI = 1.09–2.01) and a significantly higher risk of the CC and CT genotypes for Caucasians (OR = 1.26, 95% CI = 1.06–1.50). Additionally, the funnel plot results were balanced, with the *p*-value for Egger’s test greater than 0.05.

**Fig 3:**
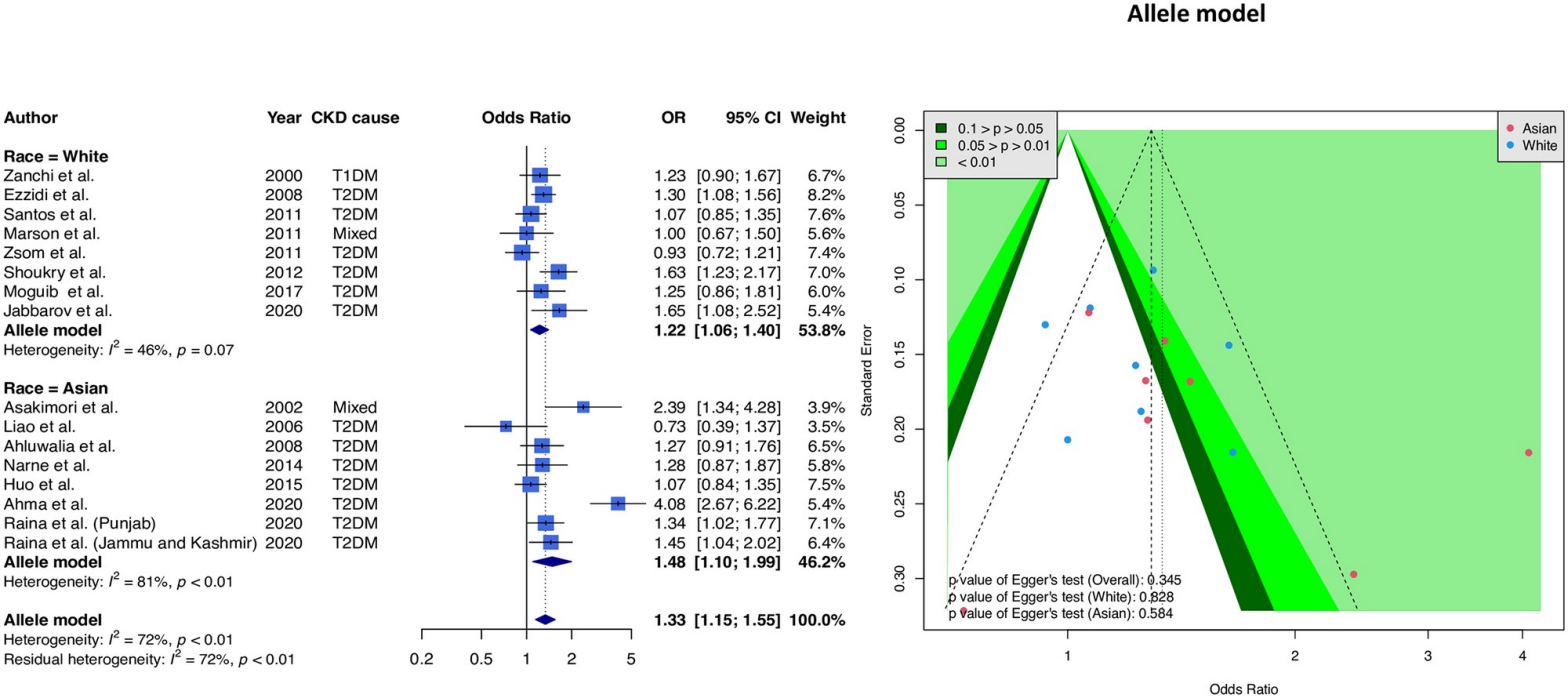
Forest and funnel plots of the relationship between eNOS T-786C alleles and CKD (allele model). The forest plot is based on the allele model assumption (reference: T allele), and the funnel plot is based on the allele model assumption. The allele model is the most common method for detecting gene-disease associations; we found a significant association among Asian and White subgroups. However, the funnel plot indicated balanced symmetry in this meta-analysis.

We then use the allele model (C allele *vs*. T allele) to combine 16 samples. Fig 3 shows the forest plot and funnel plot. The combined result of all articles shows a significantly higher risk of the TT genotype (OR = 1.33, 95% CI = 1.15–1.55). Ethnicity-stratified results showed a significant association for Asians (OR = 1.48, 95% CI = 1.10–1.99) and a significantly higher risk of the TT genotype for Caucasians (OR = 1.22, 95% CI = 1.06–1.40). Additionally, the funnel plot results were balanced, with the *p*-value for Egger’s test greater than 0.05.

**Fig 4:**
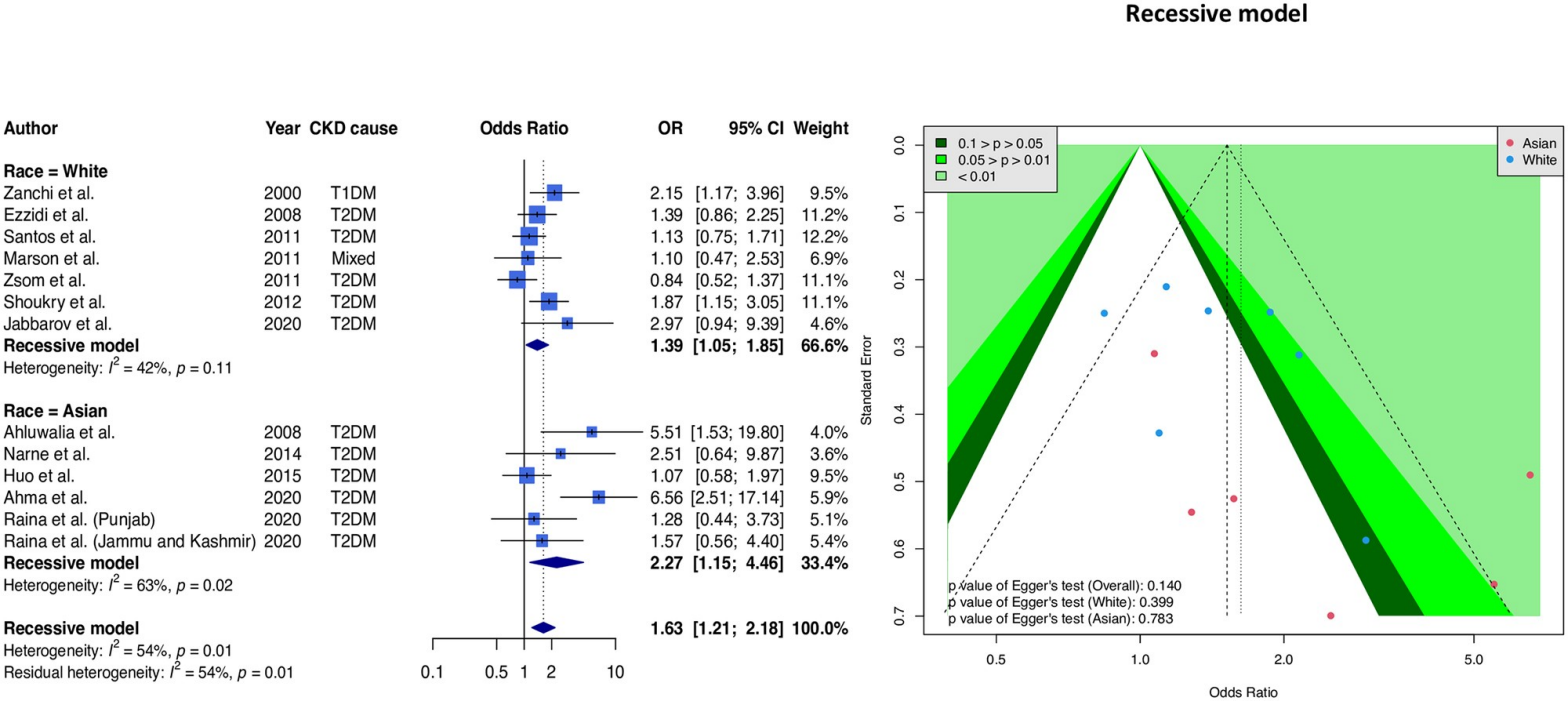
Forest and funnel plots of the relationship between eNOS T-786C alleles and CKD (recessive model). The forest plot is based on a recessive model assumption (reference: CT + CC genotype), and the funnel plot is based on the recessive model assumption; we found a significant association among Asian and White subgroups. However, the funnel plot indicated balanced symmetry in this meta-analysis.

Finally, we used the recessive model (C/C vs. C/T or T/T genotype) to combine 16 samples. Fig 4 shows the forest plot and funnel plot. Combined results of all articles showed a significantly higher risk of the CC genotype (OR = 1.63, 95% CI 1.21–2.18). Ethnicity-stratified results show significant association for Asians (OR = 2.27, 95% CI = 1.15–4.46) and a significantly higher risk of the CC genotype for Caucasians (OR = 1.39, 95% CI = 1.05–1.85). Additionally, the funnel plot results were balanced, with the *p*-value for Egger’s test being greater than 0.05.

From the above results, the overall results are significant. Then, we used TSA to test whether the sample size is large enough to reach a definite conclusion.

### 3.2 TSA sample estimation

**Fig 5:**
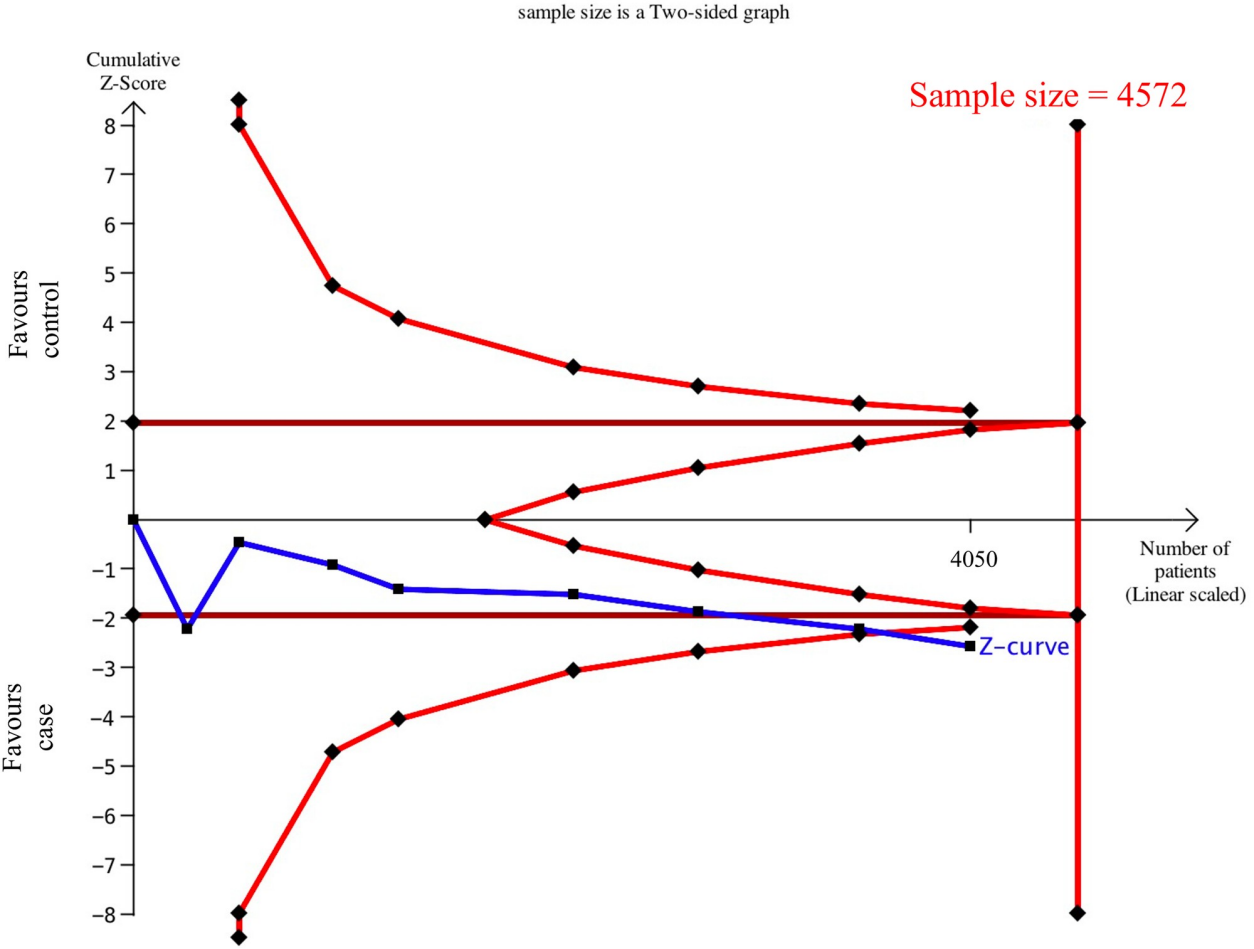
Estimation of the Asian sample size for the relationship between eNOS T-786C and CKD. TSA is a methodology that includes a sample size calculation for a meta-analysis with the threshold of statistical significance. We performed a TSA using an allele model assumption but replaced the allele count with the sample size (divided by 2). Detailed settings: Significance level = 0.05; power = 0.80; ratio of controls to cases = 1; hypothetical proportion of C allele in control = 0.10; least extreme OR to be detected = 1.5; I^2^ (heterogeneity) = 70%.

After TSA estimation, the cumulative sample size for Caucasians was 3794 patients (S1 Fig). No significant relationship was found between T-786C and CKD, and the cumulative sample size has exceeded the target sample size (n = 1030). Therefore, definite results can be obtained for Caucasians. The cumulative sample size for Asians was 4050 patients (Fig 5), and there was a significant relationship between eNOS T-786C and CKD. However, the cumulative sample size did not reach the target sample size yet (n = 4572). Therefore, definite results could not be obtained for Asians. Therefore, we added our case-control study (1198 patients) to further expand the sample and thus validate the association.

### 3.3 Case-study control

Table 1 shows the distribution of the general demographic variables and blood biochemistry values of the case-control population. We recruited 1198 subjects in this study, including 640 subjects in the control group with a mean age of 72.49 ± 6.96 years (213 men and 427 women) and 558 subjects in the case group with a mean age of 64.50 ± 14.97 years (255 men and 303 women). The proportion of male subjects and the mean age in the control group were lower than those in the case group (*p* < 0.001). Compared with the control group, the case group had a higher prevalence of diabetes and hypertension and higher creatinine and triglyceride levels. Additionally, the case group had lower cholesterol levels and glomerular filtration rates. Table 2 shows the distribution differences in the eNOS T-786C genotype between the case and control groups. The genotype frequency of the control group did not deviate from the Hardy–Weinberg equilibrium (p = 0.329). The frequency of the C allele did not show any significant differences between the control and case groups (p = 0.193). Table 3 shows that eNOS T-786C is not significantly correlated with ESRD [OR 1.01 (95% CI = 0.69–1.46). We further used different genetic models to validate the results and similarly did not find any significant results. Therefore, our case-control study found that eNOS T-786C did not have a significant association with ESRD. To increase the level of evidence for the meta-analysis, we pooled samples of case-control studies into the meta-analysis and further employed TSA validation to see if definite conclusions could be obtained for the Asian population.

**Table 1:** Distribution of general demographic variables and blood biochemical tests in case-control populations.

	Control (N = 640)	ESRD (N = 558)	P-value
Males (%)	213(35.6%)	255(45.6%)	<0.001*
Age (mean ± SD)	72.49 ± 6.96	64.50 ± 14.97	<0.001*
BMI (mean ± SD)	24.08 ± 3.90	22.48 ± 4.00	<0.001*
Diabetes (%)	70(14.5%)	191(56.0%)	<0.001*
Hypertension (%)	182(37.7%)	285(56.5%)	<0.001*
Blood biochemistry tests (mean ± SD)			
Cholesterol, mg/dl	187.01 ± 31.93	166.15 ± 36.16	<0.001*
Triglycerides, mg/dl	113.47 ± 79.29	157.97 ± 107.68	<0.001*
Creatine, mg/dl	0.72 ± 0.12	9.54 ± 2.53	<0.001*
Estimated glomerular filtration rate, mL/min/1.73 m^2^	97.06 ± 12.92	5.54 ± 1.90	<0.001*

Control group: Subjects with glomerular filtration rate > 60; ESRD group: Hemodialysis patients with glomerular filtration rate < 15.

*p < 0.05.

**Table 2:** eNOS T-786C genotype distribution in the ESRD group and control group.

Genotype	Control (N = 640)	ESRD (N = 558)	p-value
Allele			0.193
T allele	1135(88.7%)	904(81.0%)	
C allele	145(11.3%)	212(19.0%)	
Co-dominant			0.327
TT	507(79.2%)	452(81.0%)	
CT	121(18.9%)	101(18.1%)	
CC	12(1.9%)	5(0.9%)	
Dominant			0.441
TT	507(79.2%)	452(81.0%)	
CT + CC	133(20.8%)	106(19.0%)	
Recessive			0.153
TT + CT	628(98.1%)	553(99.1%)	
CC	12(1.9%)	5(0.9%)	

*p < 0.05.

**Table 3:** Results of the analysis of the relationship between eNOS T-786C gene polymorphism and ESRD.

	Crude-OR (95% CI)	p-value	Adj-OR (95% CI)#	p-value
Allele				
T allele	1		1	
C allele	0.86 (0.62–1.19)	0.363	1.01 (0.69–1.46)	0.978
Co-dominant				
TT	1		1	
CT	1.06 (0.38–3.00)	0.908	0.89 (0.25–3.14)	0.857
CC	1.25 (0.45–3.47)	0.665	0.89 (0.26–3.10)	0.86
Dominant				
TT	1		1	
CT + CC	0.85 (0.66–1.09)	0.193	1.00 (0.75–1.33)	0.999
Recessive				
TT + CT	1		1	
CC	0.82 (0.30–2.28)	0.71	1.12 (0.32–3.87)	0.859

^#^Corrected for sex and age.

### 3.4 Meta-analysis results and TSA sample estimation after inclusion of the case-control study

**Fig 6:**
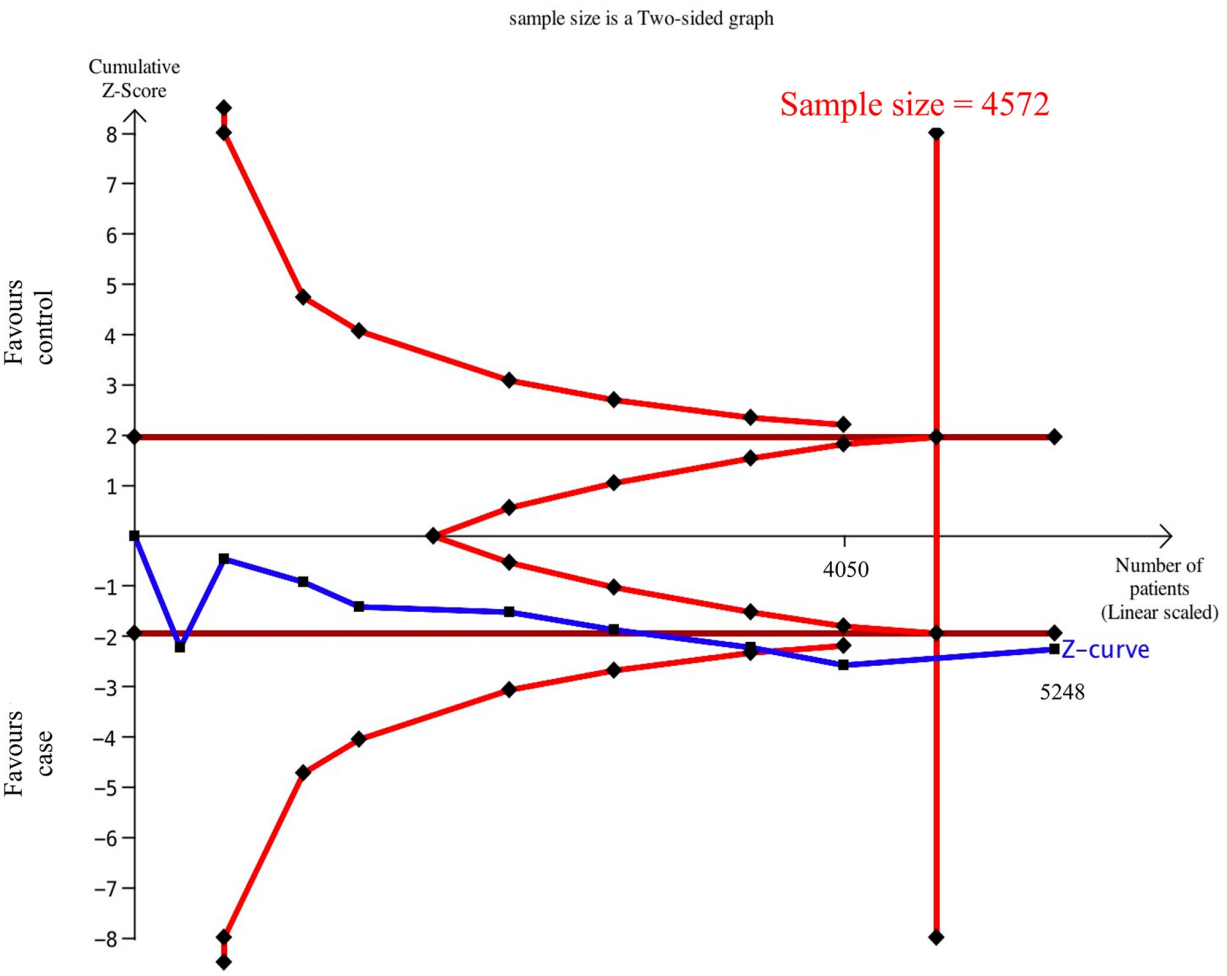
Estimation of Asian sample size for the relationship between eNOS T-786C and CKD (including this study).

**Fig 7:**
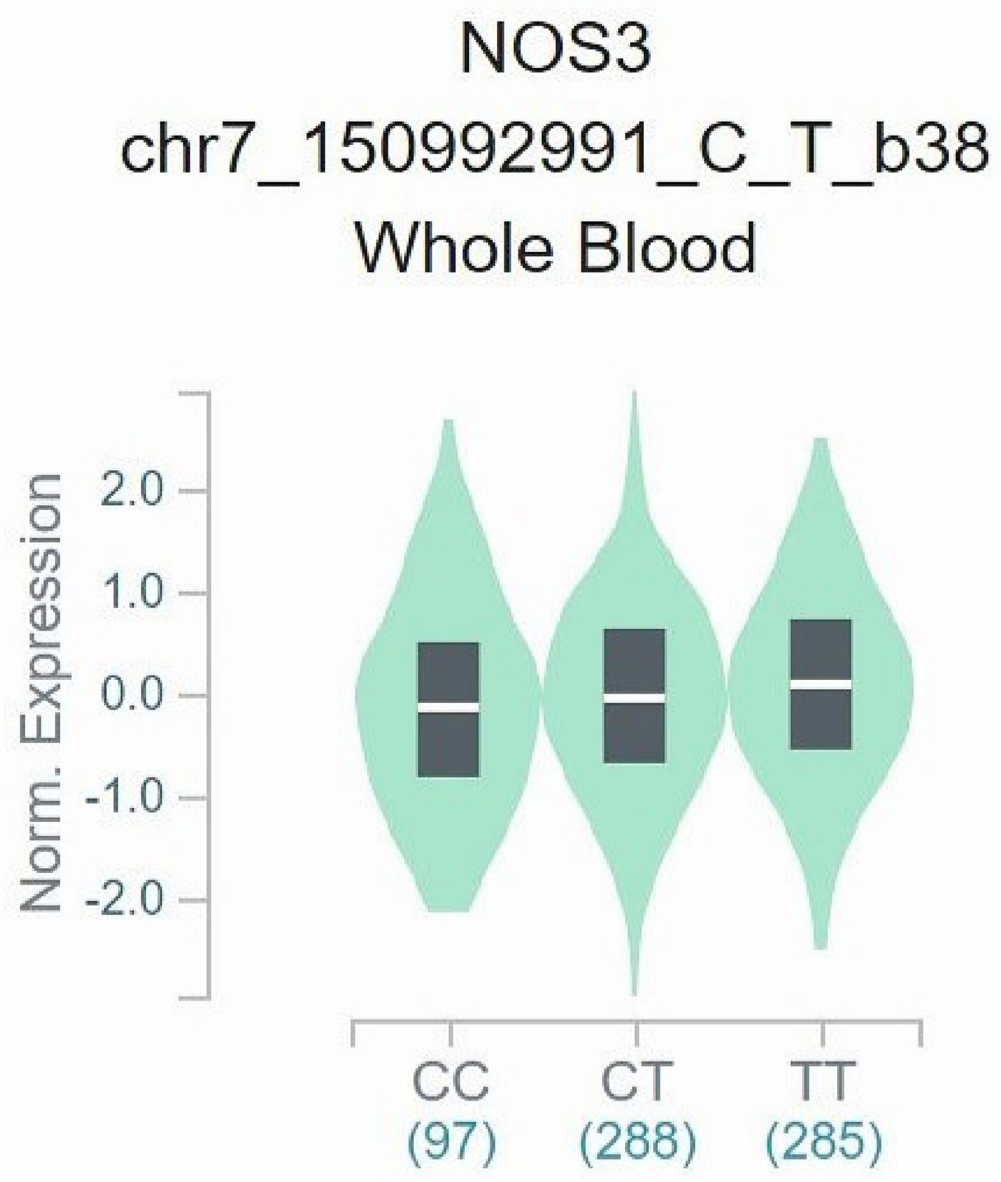
GTEx eQTL violin plot of eNOS T-786C.

After adding our case-control study, nine samples were combined in the dominant/recessive/allele model, and the results are shown below [dominant OR = 1.39 (95% CI = 1.04–1.85), recessive OR = 1.83 (95% = CI 0.92–3.64), allele OR = 1.39 (95% CI = 1.04–1.84)]. There was no hidden publication bias in the meta-analysis, according to the funnel plots (S2–S4 Figs). We employed TSA to estimate the sample size of Asians after pooling our case-control study (n = 1198) (Fig 6). Results showed that the cumulative sample size for Asians was 5248 patients, and a significant relationship was found between eNOS T-786C and CKD. The cumulative sample size curve (Z curve) has entered the futility area and exceeded the sample size of the TSA estimation (n = 4572), which means that the cumulative sample size is large enough to obtain a definite conclusion of the relationship between eNOS T-786C and CKD in Asians. Our case-control study was crucial as it turned the results from inconclusive to conclusive. Data from the GTEx project [29] showed that the C allele of T-786C was associated with decreased eNOS gene expression in the whole blood (*p* = 0.026) (Fig 7).

## 4. Discussion

This case-control study did not find any significant relationships between T-786C and ESRD risk. This result is consistent with the findings of some previous studies [20,21]. However, other studies have highlighted that T-786C is associated with ESRD risk [30,31]. Ahluwalia *et al*. showed that the C/C genotype is a risk factor for type 2 diabetic nephropathy. The possible mechanism is that the C/C genotype in diabetics will affect eNOS promoter activity, resulting in lower eNOS mRNA expression and significantly reduce NO concentrations [32]. In this study, the definition of subjects included in the case group is relatively broad. In addition to diabetic nephropathy, we included hypertension and renal parenchymal inflammation, which may result in differences in the results.

Asakimori *et al*. revealed that dialysis patients have a significantly higher frequency of the C/C or C/T genotypes than healthy controls [33]. Additionally, similar results were obtained when the cause of dialysis was divided into diabetic nephropathy or nondiabetic renal disease. The possible mechanism is that the C allele mutation at the T-786C locus causes systemic or intraglomerular hypertension and increased glomerular pressure, which is an essential factor for glomerulosclerosis. In this study, ESRD patients were divided based on different etiologies, and we examined the presence of differences in gene frequencies between the ESRD and healthy control groups. Results revealed no significant differences in the distribution (S5 Table). In the future, more studies would be needed to verify whether T-786C is associated with ESRD with different etiologies.

The decline of NO bioavailability, a hallmark of CKD, mediated by NOS3, reduces diuresis and natriuresis [34,35]. Additionally, the reduction of NO in a mouse model lead to a higher renovascular pressure, enhanced effects of angiotensin II, and deteriorated diabetic nephropathy, proteinuria, and glomerulosclerosis [36]. Data of eQTL from GTEx showed that T-786C (chr7_150992991_C_T_b38) with the C minor allele exhibited relatively lower NOS3 mRNA expression in whole blood, indicating disequilibrium of NOS3 regulating NO [29,37]. Additionally, N4-acetylcytidine (ac4C) modification of mRNA enhances the stability of mRNA and efficiency, which correlates with the occurrence, development, and prognosis of diseases. For example, escalating ac4C levels in patients with uremia show increased inflammatory responses [38]. Thus, more investigations on eNOS and ac4C would help clarify the role of eNOS in the mechanisms of CKD.

Previous meta-analyses have found that eNOS T-786C is associated with higher diabetic nephropathy risk [17,18,39]. This study used dominant, allele, and recessive models to combine results under the assumption of the random-effects model and found that eNOS T-786C was significantly associated with CKD. Furthermore, after ethnicity stratification, eNOS T-786C was significantly associated with CKD in Caucasian and Asian populations.

In the precision medicine era, genomic and environmental factors that are significantly related to disease phenotypes may be used as biomarkers for risk prediction and early diagnosis of the disease [40–46]. Well-trained deep-learning models based on these biomarkers have been widely used as tools to identify diseases [47]. Our next goal is to use deep-learning approaches to predict CKD or its progression based on genomic variants.

Our study has two major strengths. (1) We employed TSA estimation, in which previous meta-analyses have not been performed on this topic, and found that definite conclusions for eNOS T-786C and CKD can be obtained in the Caucasian population. Additionally, TSA showed that the sample size of the Asian population was originally insufficient, but a definite conclusion could also be achieved after pooling case-control samples. (2) Applying the random-effects model helps us avoid serious errors caused by the model selection based merely on heterogeneity [48]. However, some limitations in our study must be noted. (1) Non-English articles were not included in the meta-analysis, resulting in a bias of combined results. (2) The inference of susceptibility to CKD on only one single nucleotide polymorphism (SNP) within the *eNOS* gene might be limited, and the use of all TagSNPs on the *eNOS* gene would help further understand the genetic effect of eNOS on CKD. However, data on eQTL from GTEx is illustrated as our support for results. Besides, when more studies on other TagSNPs on the *eNOS* gene are considered in the meta-analysis, the issues, such as publication bias from smaller-scale analysis or heterogeneity, can be assessed or processed by using quality assessment score [49–54] or stratification [55]. (3) This study shows limited causality by only using GTEX to explore the effect of genetic variants on CKD development. In the future, linkage disequilibrium score and Mendelian randomization should be employed to detect whether the eNOS T-786C genetic polymorphism or other SNPs in this gene may causally trigger CKD development by mediating the expression of this gene in specific tissues [56–59].

Researchers usually use their samples for analysis in observational studies, while meta-analyses only deal with summarized data of published papers. In our study, we combined conventional observational studies and integrated techniques of meta-analysis to upgrade the level of evidence further. We also used TSA to verify that the sample size for Asians is sufficient to confirm the association of eNOS T-786C gene polymorphisms with CKD, attributed to our case-control samples. The sources of heterogeneity in this meta-analysis and the effects of gene-environment interactions on the risk of disease development should be further investigated in the future.

## Supporting information

S1 Fig Estimation of the Caucasian sample size for the analysis of the relationship between eNOS T-786C and CKD. (TIF)

S2 Fig Forest and funnel plots of the relationship between eNOS T-786C and CKD in a dominant gene model (including this study). (TIF)

S3 Fig Forest and funnel plots of the relationship between eNOS T-786C and CKD in an allele model (including this study). (TIF)

S4 Fig Forest and funnel plots of the relationship between eNOS T-786C and CKD in a recessive model (including this study). (TIF)

S1 Table PRISMA 2020 checklist. (DOCX)

S2 Table Search strategies and detailed records. (DOCX)

S3 Table General description of papers included in the meta-analysis. (DOCX)

S4 Table Extracted information from papers included in the meta-analysis. (DOCX)

S5 Table eNOS T-786C genotype distribution in the different ESRD etiology groups and control group. (DOCX)
