# Supplementary figures and images for: Association between nitric oxide synthase T-786C genetic polymorphism and chronic kidney disease: Meta-analysis incorporating trial sequential analysis
Source: PLoS One. 2021 Oct 18;16(10):e0258789. doi: 10.1371/journal.pone.0258789 (PMC8523046; doi:10.1371/journal.pone.0258789)

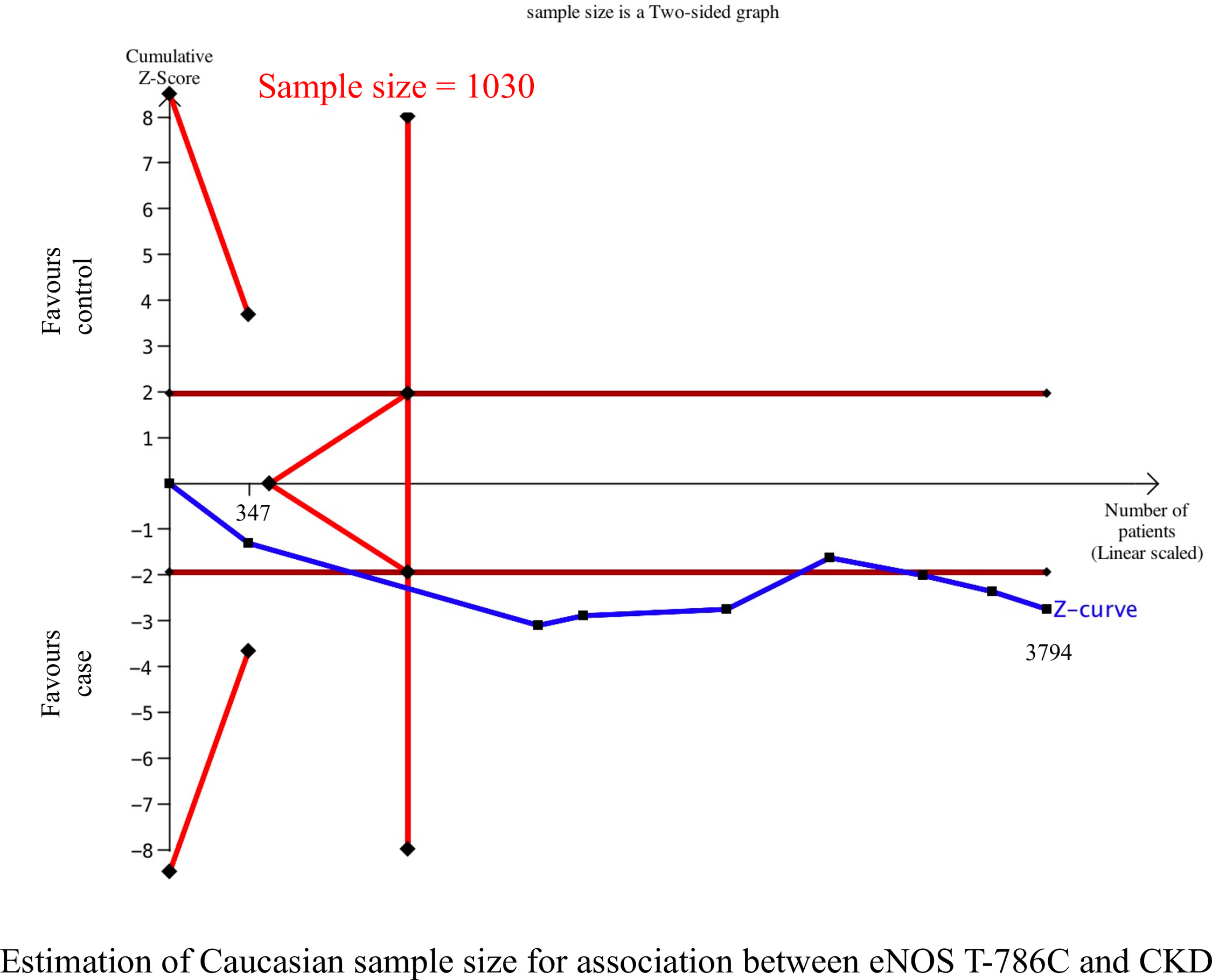

Supplement: S1 Fig — (TIF) [file pone.0258789.s001.tif]

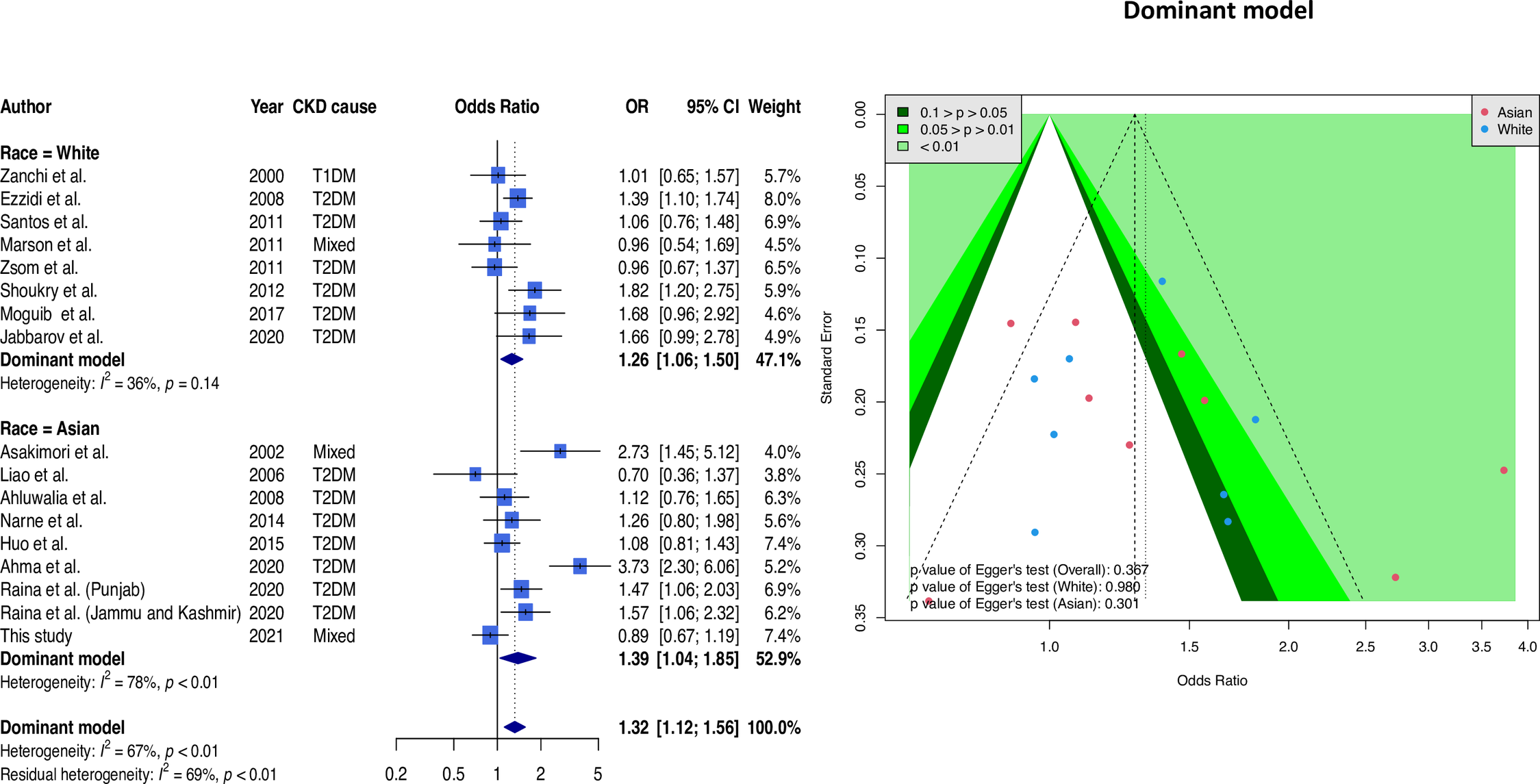

Supplement: S2 Fig — (TIF) [file pone.0258789.s002.tif]

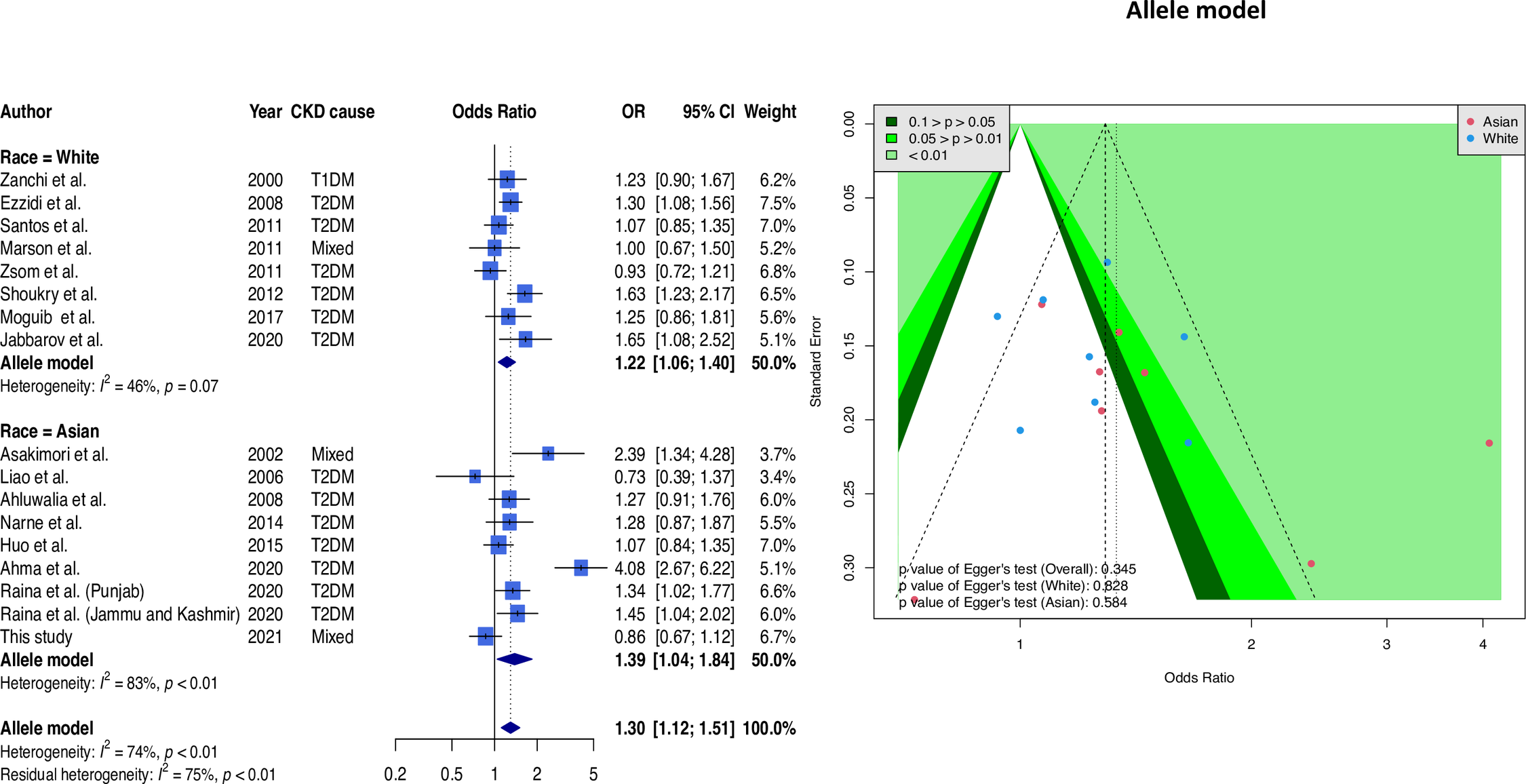

Supplement: S3 Fig — (TIF) [file pone.0258789.s003.tif]

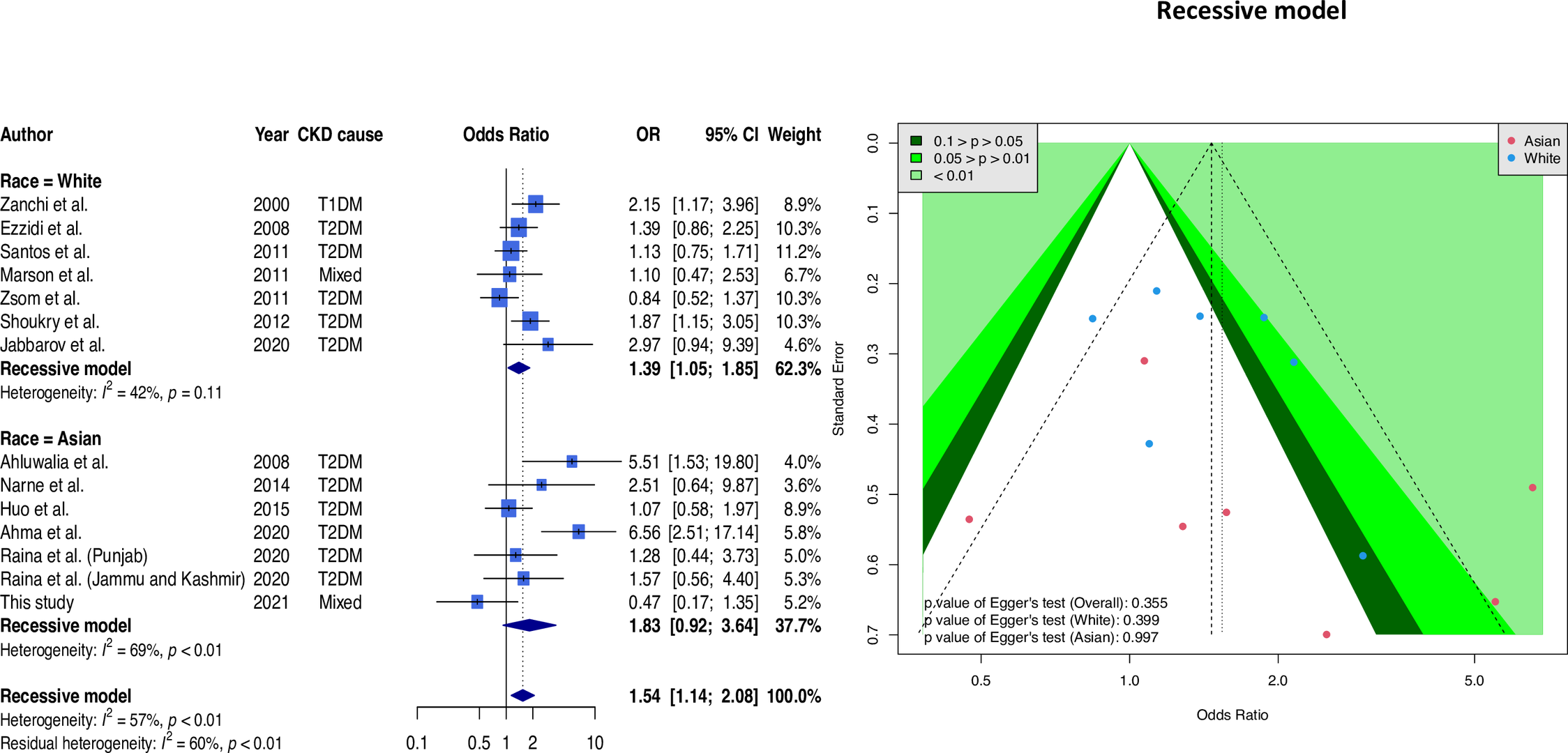

Supplement: S4 Fig — (TIF) [file pone.0258789.s004.tif]
