# Supplementary material for: Association between nitric oxide synthase T-786C genetic polymorphism and chronic kidney disease: Meta-analysis incorporating trial sequential analysis
Source: PLoS One. 2021 Oct 18;16(10):e0258789. doi: 10.1371/journal.pone.0258789 (PMC8523046; doi:10.1371/journal.pone.0258789)
Supplement: S2 Table — (DOCX) [file pone.0258789.s006.docx]

S2 Table. Search strategies and detailed records

| **Relevant text of eNOS T-786C**   1. Nitric Oxide Synthase Type III 2. ECNOS Enzyme 3. ENOS Enzyme 4. Endothelial Constitutive Nitric Oxide Synthase 5. Endothelial Nitric Oxide Synthase 6. Nitric Oxide Synthase, Type III 7. T-786C 8. polymorphisms 9. variant 10. SNP 11. rs2070744 12. ((1 or 2 or 3 or 4 or 5 or 6 ) and ((7 or 8 or 9 or 10)) or 11   **Relevant text of chronic kidney disease**   1. Renal Insufficiency, Chronic 2. Chronic kidney disease 3. Chronic Kidney Insufficiency 4. Chronic Renal Diseases 5. Chronic Renal Insufficiency | 1. Kidney Insufficiency, Chronic 2. CKD 3. Kidney Failure, Chronic 4. Chronic Kidney Failure 5. End-Stage Kidney Disease 6. End-Stage Renal Disease 7. End-Stage Renal Failure 8. Renal Disease, End-Stage 9. Renal Failure, Chronic 10. Renal Failure, End-Stage 11. ESKD 12. ESRD 13. ESRF 14. Proteinuria 15. Albuminuria 16. Nephropathy 17. Nephritis 18. Dialysis 19. Glomerular filtration rate 20. GFR 21. 13 or 14 or 15 or 16 or 17 or 18 or 19 or 20 or 21 or 22 or 23 or 24 or 25 or 26 or 27 or 28 or 29 or 30 or 31 or 32 or 33 or 34 or 35 or 36 or 37   **Combined (Final strategy)**   1. 12 and 38 |
| --- | --- |

Web sites and uniform resource locator :

**MeSHBrowser**: <http://www.nlm.nih.gov/mesh/MBrowser.html>

**PubMed**: <http://www.ncbi.nlm.nih.gov/pubmed>

**Embase**: https://www.embase.com
