# Supplementary material for: Association between nitric oxide synthase T-786C genetic polymorphism and chronic kidney disease: Meta-analysis incorporating trial sequential analysis
Source: PLoS One. 2021 Oct 18;16(10):e0258789. doi: 10.1371/journal.pone.0258789 (PMC8523046; doi:10.1371/journal.pone.0258789)
Supplement: S3 Table — (DOCX) [file pone.0258789.s007.docx]

S3 Table. General description of papers included in meta-analysis

| **Author** | **Year** | **Country** | **Ethnicity** | **Study**  **design^a^** | **CKD type^b^** | **Kidney function**  **of case** ^c^ | **Definition of case group** ^d^ |
| --- | --- | --- | --- | --- | --- | --- | --- |
| Moguib | 2017 | Egypt | Caucasian | CS | T2DN | non-ESRD | albuminuria |
| Huo | 2015 | China | Asian | CC | T2DN | non-ESRD | urinary albumin>500 mg/L or ACR>300 mg/g |
| Narne | 2014 | India | Asian | CC | T2DN | non-ESRD | UAE>300 mg/24h |
| Shoukry | 2012 | Egypt | Caucasian | CC | T2DN | non-ESRD | ACR >300mg/g |
| Santos | 2011 | Brazil | Caucasian | CC | T2DN | non-ESRD | Increased UAE or dialysis |
| Marson | 2011 | Brazil | Caucasian | CC | Mixed | ESRD | HD or PD |
| Zsom | 2011 | Hungary | Caucasian | CC | T2DN | ESRD | HD |
| Ezzidi | 2008 | Tunisia | Caucasian | CC | T2DN | non-ESRD | AER>30 mg/24h or plasma creatinine>176 μmol/L |
| Ahluwalia | 2008 | India | Asian | CC | T2DN | non-ESRD | UAE > 500mg/l or ACR >300mg/g |
| Liao | 2006 | China | Asian | CC | T2DN | non-ESRD | AER>30 mg/24 h |
| Asakimori | 2002 | Japan | Asian | CC | Mixed | ESRD | HD |
| Zanchi | 2000 | USA | Caucasian | CC | T1DN | non-ESRD | Proteinuria or HD or RRT |
| This Study | 2018 | Taiwan | Asian | CC | Mixed | ESRD | HD |

^a^: CC – case control study; CS – cross sectional study.

^b^: T1DN – Type 1 diabetic nephropathy; T2DN –Type 2 diabetic nephropathy; Mixed –Diabetic nephropathy, hypertensive nephropathy^c^:,etc ESRD – only ESRD patients; non-ESRD – not only ESRD patients.

^d^: ACR – albumin creatinine ratio; AER – albumin excretion rate; UAE –urinary albumin excretion rate; eGFR – estimated glomerular filtration rate; HD – hemodialysis; CCr – creatinine clearance; RRT – renal replacement therapy; CT – computed tomography; SCr – serum creatinine
