# Supplementary material for: Association between nitric oxide synthase T-786C genetic polymorphism and chronic kidney disease: Meta-analysis incorporating trial sequential analysis
Source: PLoS One. 2021 Oct 18;16(10):e0258789. doi: 10.1371/journal.pone.0258789 (PMC8523046; doi:10.1371/journal.pone.0258789)
Supplement: S4 Table — (DOCX) [file pone.0258789.s008.docx]

S4 Table. Extracted information from papers included in meta-analysis

| **Author** | **Year** | **Quality^a^** | **Sex^b^** | **BMI^c^** | **HT^d^** | **DM^e^** | **Case group** | | | **Control group** | | |
| --- | --- | --- | --- | --- | --- | --- | --- | --- | --- | --- | --- | --- |
|  |  |  |  |  |  |  | **TT** | **CT** | **CC** | **TT** | **CT** | **CC** |
| Moguib | 2017 | 5 | 43 | 32.1 | 67 | 100 | 37 | 126 | 0 | 33 | 67 | 0 |
| Huo | 2015 | 3 | 51 |  | 55.7 | 100 | 280 | 128 | 23 | 280 | 119 | 21 |
| Narne | 2014 | 5 | 63.2 | 28.6 | 80.6 | 100 | 89 | 59 | 7 | 102 | 57 | 3 |
| Shoukry | 2012 | 2 | 54 | 27.4 | 50.2 | 100 | 57 | 89 | 54 | 84 | 83 | 33 |
| Santos | 2011 | 7 | 57.2 | 28.7 | 85.2 | 100 | 140 | 160 | 76 | 93 | 104 | 44 |
| Marson | 2011 | 4 | 72 | 25.9 | 76 | 24 | 39 | 48 | 13 | 38 | 50 | 12 |
| Zsom | 2011 | 3 | 56.4 |  | 15.3 | 34.2 | 130 | 157 | 46 | 76 | 92 | 32 |
| Ezzidi | 2008 | 7 | 46 | 27.4 | 44.7 | 100 | 261 | 215 | 34 | 436 | 264 | 36 |
| Ahluwalia | 2008 | 4 | 35 | 28.01 | 50.9 | 100 | 121 | 62 | 12 | 165 | 87 | 3 |
| Liao | 2006 |  |  |  |  |  | 138 | 24 | 0 | 77 | 19 | 0 |
| Asakimori | 2002 | 3 | 50 |  |  | 100 | 50 | 24 | 0 | 159 | 28 | 0 |
| Zanchi | 2000 | 2 | 49 |  | 49.8 | 100 | 58 | 64 | 30 | 75 | 100 | 20 |
| This Study | 2011 | 9 | 64.5 | 22.48 | 56.5 | 56 | 452 | 101 | 5 | 507 | 121 | 12 |

^a^: Literature quality, score; ^b^: Male ratio, percentage; ^c^: Body Mass Index, kg/m^2^; ^d^: Prevalence of Hypertension, percentage.^e^: Prevalence of Diabetes mellitus, percentage
