# Supplementary material for: Association between nitric oxide synthase T-786C genetic polymorphism and chronic kidney disease: Meta-analysis incorporating trial sequential analysis
Source: PLoS One. 2021 Oct 18;16(10):e0258789. doi: 10.1371/journal.pone.0258789 (PMC8523046; doi:10.1371/journal.pone.0258789)
Supplement: S5 Table — (DOCX) [file pone.0258789.s009.docx]

S5 Table. eNOS T-786C genotype distribution in the different ESRD etiology groups and control group

| Genotype | Control | ESRD | | | | | |
| --- | --- | --- | --- | --- | --- | --- | --- |
|  | (N *=* 640) | Parenchymal renal disease  (N=) | p-value | Diabetic nephropathy  (N=231) | p-value | Hypertensive  nephropathy  (N=69) | p-value |
| Allele |  |  | 0.219 |  | 0.672 |  | 0.676 |
| T allele | 1135(88.7%) | 304(91.0%) |  | 413(89.4%) |  | 124(89.9%) |  |
| C allele | 145(11.3%) | 30(9.0%) |  | 49(10.6%) |  | 14(10.1%) |  |
| Co-dominant |  |  | 0.326 |  | 0.889 |  | 0.924 |
| TT | 507(79.2%) | 138(82.6%) |  | 184(79.7%) |  | 55(79.7%) |  |
| CT | 121(18.9%) | 28(16.8%) |  | 45(19.5%) |  | 14(20.3%) |  |
| CC | 12(1.9%) | 1(0.6%) |  | 2(0.9%) |  | 0(0.0%) |  |
| Dominant |  |  | 0.457 |  | 0.652 |  | 0.741 |
| TT | 507(79.2%) | 138(82.6%) |  | 184(79.7%) |  | 55(79.7) |  |
| CT + CC | 133(20.8%) | 29(17.4%) |  | 47(20.3%) |  | 14(20.3) |  |
| Recessive |  |  | 0.487 |  | 0.376 |  | 0.619 |
| TT + CT | 628(98.1%) | 166(99.4%) |  | 229(99.1%) |  | 69(100%) |  |
| CC | 12(1.9%) | 1(0.6%) |  | 2(0.9%) |  | 0(0%) |  |
